# Development of a Dual Gene-Targeted Multi-Sirna with Branched Structure and Its Role in the Therapy of Liver Cancer

**DOI:** 10.3390/ph18121844

**Published:** 2025-12-03

**Authors:** Mingdong Lu, Wenqin Jiang, Zhekai Liu, Yiqing Liu, Fangli Wu, Weibo Jin

**Affiliations:** 1 Key Laboratory of Plant Secondary Metabolism and Regulation of Zhejiang Province, College of Life Sciences and Medicine, Zhejiang Sci-Tech University, Hangzhou 310018, China; 15868153437@163.com (M.L.); jiangwenqin@163.com (W.J.); lzk1870123320@gmail.com (Z.L.); 2023210901042@mails.zstu.edu.cn (Y.L.); wfl@zstu.edu.cn (F.W.); 2 Zhejiang Sci-Tech University Shaoxing Academy of Biomedicine, Shaoxing 312366, China

**Keywords:** multi-siRNA, branched structure, RNAi therapy, liver cancer, *GP73*, *hTERT*

## Abstract

**Background:** Hepatocellular carcinoma (HCC) remains a major global health challenge with limited therapeutic options. Although RNA interference (RNAi) enables precise gene silencing, its clinical application is restricted by siRNA instability, inefficient cellular uptake, and the requirement for potentially toxic delivery carriers. To address these limitations, a dual-targeted branched siRNA nanostructure (GT-multi-siRNA) was developed to simultaneously silence two HCC-related oncogenes, GP73 and hTERT. **Methods:** GT-multi-siRNA was synthesized in *Escherichia coli* and characterized for particle size, stability, Dicer processing efficiency, intracellular retention, and cytotoxicity. Its therapeutic effects were evaluated through gene-silencing assays, proliferation and migration assays in Hep3B cells, and intratumoral administration in a xenograft mouse model. Histopathology and cytokine profiling were conducted to assess biosafety. **Results:** GT-multi-siRNA formed uniform nanoparticles (50–100 nm) with moderate physicochemical stability and minimal cytotoxicity at concentrations ≤ 200 ng/μL. The nanostructure was efficiently processed by Dicer into functional siRNAs and remained detectable intracellularly for at least 36 h. In Hep3B cells, GT-multi-siRNA reduced GP73 and hTERT mRNA and protein levels by approximately 50%, accompanied by significant inhibition of cell proliferation and migration. In vivo, a single intratumoral dose suppressed tumor growth, while a two-dose regimen markedly limited tumor progression. No liver toxicity was observed, and cytokine analysis showed selective IL-4 upregulation without influencing IL-6 levels. **Conclusions:** GT-multi-siRNA demonstrates potent dual-gene silencing activity and favorable biosafety, providing a promising RNAi-based therapeutic strategy for targeted HCC treatment.

## 1. Introduction

Cancer remains a leading cause of morbidity and mortality worldwide, with hepatocellular carcinoma (HCC) representing the most common form of primary liver cancer and the third leading cause of cancer-related death globally [1,2]. Primary liver cancer originates in the liver and is called hepatocellular carcinoma (HCC). Hepatocellular carcinoma is considered the most common primary liver cancer in adults, accounting for about 90% of primary liver cancers [3]. Hepatocellular carcinoma is the fifth most common cancer in the world, second only to pancreatic cancer, with a five-year survival rate of only 18% [4]. Conventional therapeutic options for HCC remain insufficient, and their limited efficacy highlights the urgent need for alternative strategies [5,6]. RNA interference (RNAi) has emerged as a precise gene-silencing approach with significant potential in cancer therapy [7]. Small interfering RNAs (siRNAs), as the key effector molecules of RNAi, can specifically downregulate target genes through sequence-dependent recognition [8,9]. Silencing oncogenic pathways with siRNAs can effectively suppress tumor cell growth, migration, invasion, and overall malignancy. Despite these advantages, achieving safe and efficient delivery of siRNAs into cancer cells continues to be a major obstacle [10,11]. Recent reviews have further highlighted advances in nanoparticle and nanomedicine-based RNA delivery systems [12,13,14,15]. In addition to synthetic carriers, innovative biological delivery platforms have also emerged. For example, radiolytically engineered *Escherichia coli* Nissle has recently been reported as a live-therapeutic system capable of delivering radio-sensitizing molecules directly within tumors, demonstrating the expanding potential of engineered microorganisms in cancer-targeted payload delivery [16].

High molecular weight siRNAs were proposed to improve the physical drawbacks of siRNA [17]. Compared with the rigid rod-like structure of siRNA, a more flexible chain of high molecular weight siRNA is favorable to form condensed and compact nanoparticles with cationic carriers. A long linear dsRNA was developed as a high molecular siRNA by connecting multiple siRNAs (multi-siRNA) with a cleavable and non-cleavable cross linker. Long double-stranded RNA (dsRNA) demonstrates enhanced gene-silencing efficiency relative to naked siRNA [18]. Lee et al. reported that a dual gene targeted multi-siRNA which was developed to induce simultaneous gene knockdown of two selective proteins of GFP and VEGF. Interestingly, the dual gene targeted multi-siRNA induced enhanced gene silencing of target proteins as compared with the single gene targeted multi-siRNA [19]. In addition, branched and dendrimer-like siRNA structures have been developed may provide a prolonged RNAi effect due to their increased serum stability and charge density [20,21]. Therefore, we will design a dual-targeted, branched multi-siRNA construct to improve tumor-specific delivery and gene silencing efficiency.

It is generally believed that the occurrence of tumors is related to related signaling pathways, which are intertwined, interacting, and influencing each other [22]. They jointly act on tumors and participate in regulating mechanisms such as tumor cell proliferation, invasion, metastasis, angiogenesis, and immune response [23]. Golgi protein 73 (GP73) functions as an oncogenic factor and is modulated by inflammatory pathways, various microRNAs, and aberrantly activated mTOR signaling [24]. In normal liver tissue, GP73 expression is essentially limited to bile duct epithelial cells, with hepatocytes expressing little or none of this protein [25]. A broad range of liver injuries, however, trigger pronounced GP73 upregulation within hepatocytes, while levels in biliary cells remain relatively constant. This increase has been reported in conditions such as HCV infection, HBV-associated cirrhosis and carcinoma, alcoholic liver injury, and autoimmune hepatitis [26]. Despite substantial evidence linking GP73 to hepatocarcinogenesis, no inhibitory drugs targeting this molecule have yet reached development [23].

Human telomerase reverse transcriptase (*hTERT*) is a core catalytic component of telomerase that plays a crucial role in maintaining telomere length [27]. In most somatic cells, *hTERT* expressions are suppressed. *hTERT* overexpression is detected up to 90% of cancer cells, compared to <20% of normal cells [28]. Evidence has suggested that *hTERT* overexpression in HCC is associated with poor clinical outcomes [29]. *hTERT* gene is permanently activated in liver neoplasms from the very early stage of hepatocarcinogenesis mainly through the accumulation of genetic alterations, virus-related insertional mutagenesis, and somatic mutations in the *hTERT* promoter region. Several lines of evidence suggest that telomerase, beyond the canonical function of telomeres elongation, has multiple oncogenic activities in cancer cells and may represent a promising therapeutic target in hepatocellular carcinoma [30]. Recent evidence suggests that while GP73 mainly contributes to hepatocellular carcinoma progression through facilitating epithelial–mesenchymal transition (EMT), migration, and immune evasion, hTERT primarily drives uncontrolled proliferation and cellular immortality. These two oncogenes function in complementary but interconnected pathways—GP73 activation is linked to inflammation and mTOR signaling, whereas hTERT promotes telomerase-dependent and -independent oncogenic processes. Emerging data indicate that both are often co-upregulated in advanced HCC and may act synergistically to sustain tumor aggressiveness and therapy resistance [25]. Therefore, dual silencing of GP73 and hTERT may simultaneously block the metastatic and proliferative capacities of HCC cells, offering a more effective therapeutic strategy. Therefore, a dual gene targeted multi-siRNA with a branched structure was developed in this study through connecting *GP73*- and *hTERT*-siRNAs to silence both *GP73* and *hTERT* in HCC cells and tumors. The anti-HCC efficacy and mechanisms of the multi-siRNA were systematically elucidated by a Hep3B cell and a Hep3B tumor-bearing mouse model.

## 2. Results

### 2.1. Expression of GP73 and hTERT in HCC Patients and Its Relationship with Survival Rate

**Figure 1:**
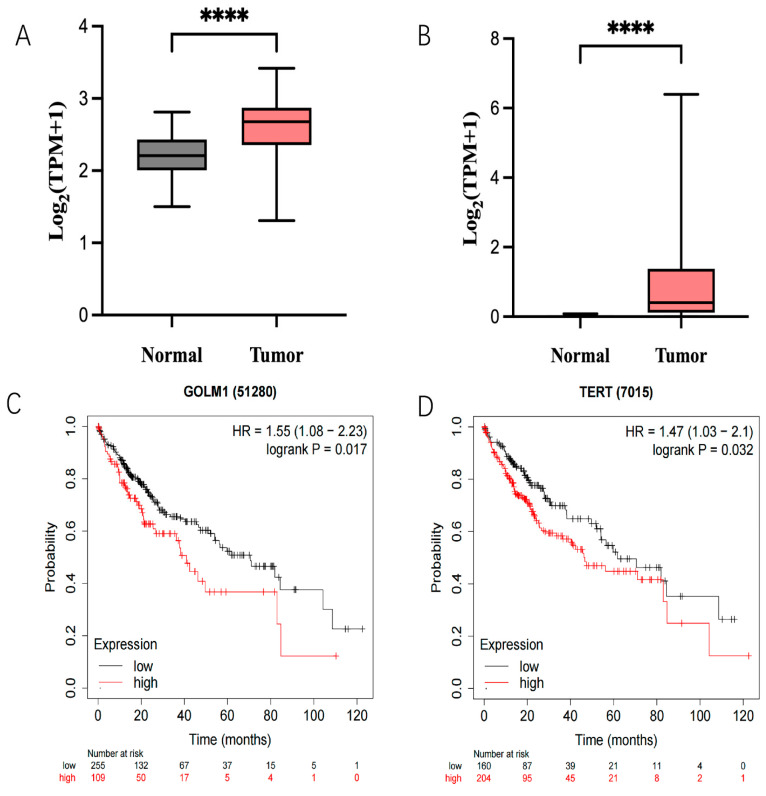
Expression of GP73 (**A**) and hTERT (**B**) in tumor tissues compared to normal tissues. Mega-sampler analysis of expression patterns about both GP73 and hTERT in cancer as well as normal tissues from TCGA dataset. (**C**) Kaplan–Meier survival curves of GP73 expression and Liver hepatocellular carcinoma patients. Statistical significance (****, *p* < 0.0001). (**D**) Kaplan–Meier survival curves of hTERT expression and Liver hepatocellular carcinoma patients. Statistical significance (****, *p* < 0.0001).

To understand the expression of *GP73* and *hTERT* in HCC and their clinical significance, the expression data of both genes in 374 HCC tissues and 50 adjacent tissues were retrieved from the TCGA-LIHC database (Table S2). Results confirmed that the expression levels of *GP73* and *hTERT* in liver cancer are much higher than those in adjacent tissues (Figure 1A,B). Moreover, low levels of *GP73* and *hTERT* can greatly improve the 5-year survival rate of HCC patients based on TCGA-LIHC clinical data (Figure 1C,D). Therefore, Both *GP73* and *hTERT* were selected as the targets to develop a polymerized branched siRNA for RNAi therapy of liver cancer.

### 2.2. Design and In Vivo Synthesis of Multi-siRNA Targeting GP73 and hTERT

**Figure 2:**
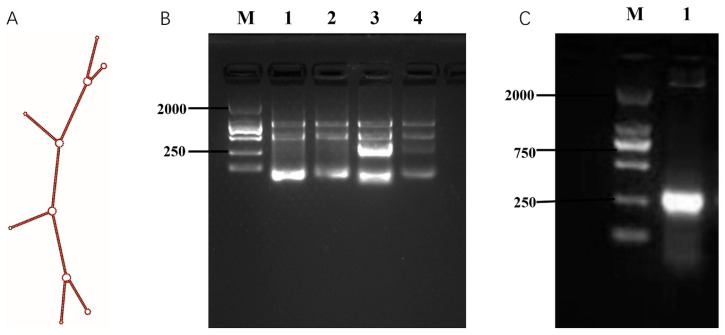
Design and biosynthesis of GT-multi-siRNA. (**A**) Secondary structure of GT-multi-siRNA predicted by RNAfold. (**B**) Expression of GT-multi-siRNA in *E. coli.* Lane M: DL2000 DNA Marker; Lane 1–2: The total RNAs of *E. coli* containing an empty pET28a plasmid with and without IPTG induction. Lane 3–4: The total RNAs extracted from the *E. coli* containing the recombinant plasmid with and without IPTG induction. (**C**) Simple extraction of GT-multi-siRNA. Lane M: DL2000; Lane 1: The specific GT-multi-siRNA was extracted by a solution containing 10 mM Tris-HCl (pH 8.0) and 10 mM MgSO_4_.

**Figure 3:**
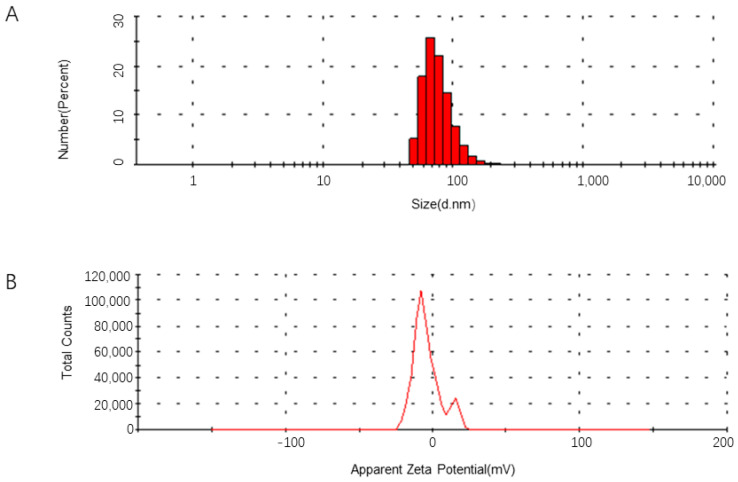
Particle size distribution of GT-multi-siRNA (**A**) and Zeta potential analysis (**B**).

To develop a polymerized branched siRNA, *GP73-* and *hTERT*-targeted siRNAs designed by DSIR were connected to form a dual gene-targeted polymerized siRNA with a branched structure. The results showed that the secondary structure of the polymerized siRNA was correct (Figure 2A) and named as GT-multi-siRNA. The GT-multi-siRNAs were then synthesized in *E. coli* and detected by running agarose gel. Results showed that the total RNAs from the *E. coli* containing an empty pET28a plasmid have only three rRNA bands, but the total RNAs extracted from the *E. coli* containing the recombinant plasmid have a specific highlight band besides three rRNA bands, indicating that GT-multi-siRNA could be synthesized in the *E. coli* with higher efficiency (Figure 2B). To extract specific GT-multi-siRNA from *E. coli*, considering the easy degradation of RNA in an alkaline solution, the original RNA extraction solution was replaced with a Tris solution containing 10 mM Tris-HCl (pH 8.0) and 10 mM MgSO_4_. The GT-multi-siRNA was extracted from IPTG-induced *E. coli* again. As expected, only GT-multi-siRNA was presented in the agarose gel, while the three rRNAs were gone (Figure 2C). DLS and zeta potential analysis showed a particle size of 50~100 nm and a zeta potential of –10 mV, suggesting moderate stability and favorable uptake potential (Figure 3A,B).

### 2.3. Verification of Dicer-Mediated Processing and siRNA Maturation

**Figure 4:**
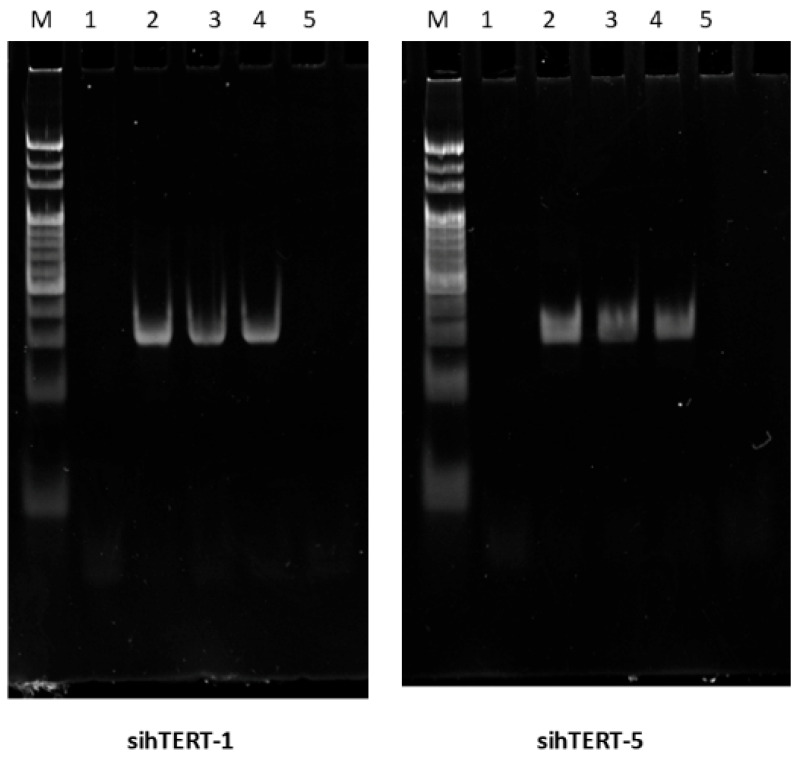
Detection of two siRNAs in GT-multi-siRNA-treated Hep3B cells. Lane M: 20 bp Ladder; Lane 1: Un-treated Hep3B cells; Lane 2–5: Hep3B cells treated with GT-multi-siRNA for 12 h, 24 h, 36 h and 48 h.

To validate that GT-multi-siRNA is processed into functional siRNAs, two siRNAs of GT-multi-siRNAs(sihTERT-1 and sihTERT-5) were detected in treated Hep3B cells at 12, 24, 36 and 48 h using stem-loop RT-PCR. The results confirmed the presence of two expected hTERT-targeted siRNAs, consistent with Dicer-mediated cleavage (Figure 3). Moreover, both siRNAs remained elevated for at least 36 h post-treatment, indicating sustained retention and activity (Figure 4).

### 2.4. Toxicity Analysis of GT-Multi-siRNA on Vero Cells

**Figure 5:**
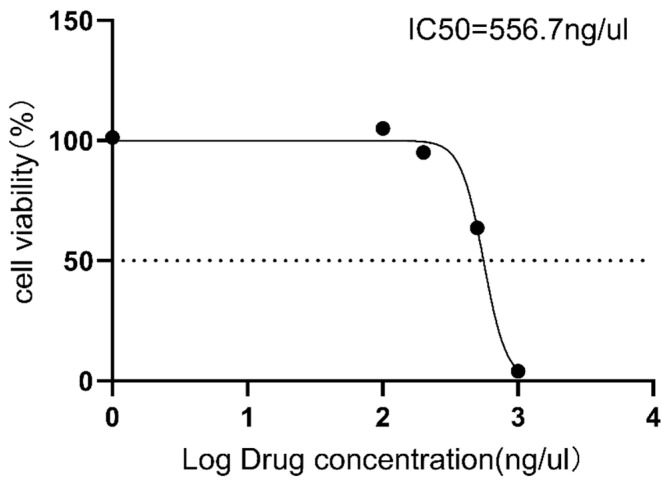
Cytotoxicity assay of GT-multi-siRNA to Vero cells. To achieve the IC50 values, Vero cells were co-incubated with increasing amounts (100 ng/μL, 200 ng/μL, 500 ng/μL, and 1000 ng/μL) of GT-multi-siRNA for 48 h. Cell viability was measured using the MTT method. The results are expressed as the means ± SDs of nine biological replicates.

To investigate the toxicity of GT-multi-siRNA, Vero cells were incubated with different concentrations of GT-multi-siRNA for 48 h. MTT assay results showed that as the concentration of GT-multi-siRNA increases, the toxicity to vero cells also increases (Figure 3 and Table S3). Moreover, the half maximal inhibitory concentration (IC50) of GT-multi-siRNA was more than 500 ng/μL, and the growth of Vero cells was not affected by GT-multi-siRNA within 200 ng/μL (Figure 5). Therefore, the concentration of GT-multi-siRNA was selected within 200 ng/μL for assessing the anti-HCC role of GT-multi-siRNA in subsequent assay.

### 2.5. Effects of GT-Multi-siRNA on the Growth of Hep3B Cell

**Figure 6:**
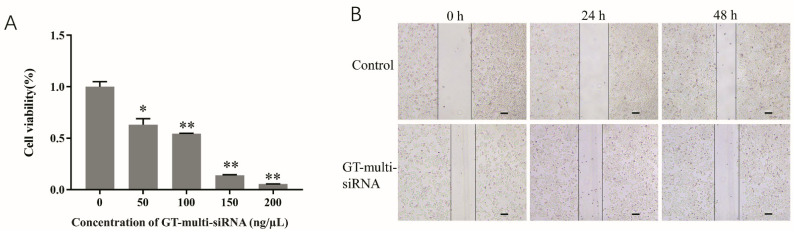
Inhibition effects of GT-multi-siRNA on the growth of Hep3B cells. (**A**) Inhibitory effect of different concentrations of GT-multi-siRNA on Hep3B proliferation using MTT method. The results are expressed as the means ± SDs of three biological replicates. Asterisks indicate a significant difference (* *p* < 0.05, ** *p* < 0.01) compared to the 0 ng/μL. (**B**) GT-multi-siRNA suppressed the migration of Hep3B cells for 0 h, 24, and 48 h by wound healing assay. The scale bar is 100 µm.

To investigate the anti-HCC activity of GT-multi-siRNA, Hep3B cells were cultured and incubated with GT-multi-siRNA for 72 h, and an MTT assay was performed to investigate the viability of Hep3B. Results showed that the survival rate of Hep3B was significantly inhibited by GT-multi-siRNA. Moreover, the inhibitory efficiency was increased with the increase of GT-multi-siRNA concentration (Figure 6A). In addition, the scratch assay also showed that the migration of Hep3B cells was inhibited by GT-multi-siRNA (Figure 6B).

### 2.6. Expressions of GP73 and hTERT in Hep3B Treated by GT-Multi-siRNA

**Figure 7:**
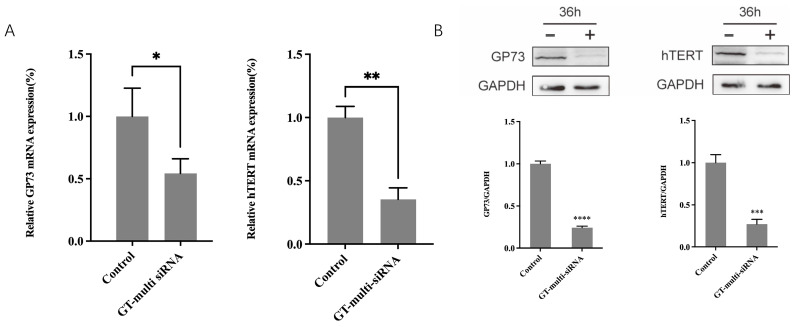
Expressions levels of GP73 and hTERT. (**A**) mRNA levels of GP73 and hTERT in Hep3B treated by GT-multi-siRNA for 24 h. (**B**) Protein levels of GP73 and hTERT in Hep3B treated by GT-multi-siRNA for 36 h. The GAPDH (NM_001256799.3) was used as the inner control. The results are expressed as the means ± SDs of three biological replicates. Asterisks indicate a significant difference (* *p* < 0.05, ** *p* < 0.01, *** *p* < 0.001, **** *p* < 0.0001) compared to the control.

To understand the mechanism of GT-multi-siRNA acts as anti-HCC, the expressions of two genes, *GP73* and *hTERT*, targeted by GT-multi-siRNA were measured by RT-qPCR and Western blot methods. RT-qPCR revealed that GT-multi-siRNA can significantly decrease the mRNA levels of both genes, and the inhibition rate was nearly 50% compared with the control (Figure 7A). Western blot showed that the protein levels of *GP73* and *hTERT* were also significantly suppressed by GT-multi-siRNA more than 50% (Figure 7B).

### 2.7. In Vivo Anti-Tumor Efficacy of GT-Multi-siRNA

**Figure 8:**
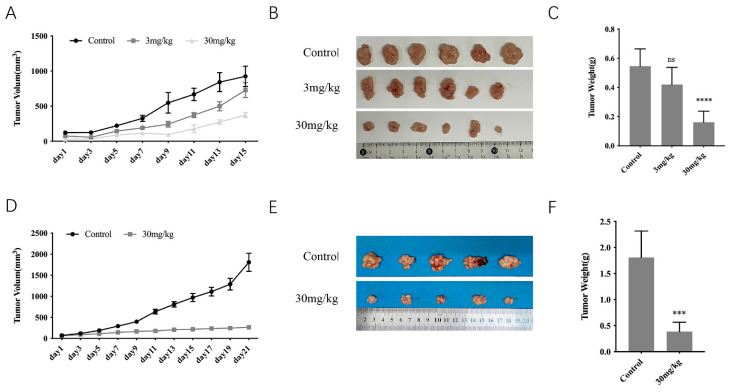
Anti-tumor effects of GT-multi-siRNA in a xenograft mouse model. (**A**–**C**) GT-multi-siRNA was injected only one time. (**A**) Tumor volume change curves. The results are expressed as the means ± SDs of six biological replicates. (**B**) The photographs of tumors in each group were removed from the sacrificed mice when the tumor volume in the control group reached approximately 1000 mm^3^. (**C**) Weights of tumors in each group. The results are expressed as the means ± SDs of six biological replicates. Asterisks indicate a significant difference (****, *p* < 0.0001; ns, not significant.) compared to the control group. (**D**–**F**) GT-multi-siRNA was injected once again at 8 dpt. (**D**) Growth curves of tumors. The results are expressed as the means ± SDs of five biological replicates. (**E**) The photographs of tumors in each group were removed from the sacrificed mice when the tumor volume in the control group reached approximately 2000 mm^3^. (**F**) Weights of tumors in each group. The results are expressed as the means ± SDs of five biological replicates. Asterisks indicate a significant difference (*** *p* < 0.001) compared to the control group.

To detect the anti-tumor potential of GT-multi-siRNA, tumor-bearing mice were administered 100 μL of PBS (control) and GT-multi-siRNA via intratumoral injection only one time. From Figure 8A, the average volume of tumors in the control group dramatically increased. However, tumor growth was inhibited by GT-multi-siRNA within 15 days post-treatment (dpt). The images of tumors obtained from mice at 15 dpt further confirmed the significant antitumor efficacy of GT-multi-siRNA (Figure 8B,C). In addition, the inhibitory effect of GT-multi-siRNA on tumor growth was better before 9 dpt than after 9 dpt, suggesting that GT-multi-siRNA should be injected again before 9 dpt to obtain sustained tumor suppression.

Next, to test the above hypothesis, the anti-tumor assay of GT-multi-siRNA was repeated using the dosage of 30 mg/kg, but instead of just one injection, the GT-multi-siRNA was injected twice. The second injection was administered at 8 dpt. The results were consistent with the hypothesis. After another injection at 8 dpt, the tumor growth was extremely slow and almost stopped (Figure 8D,E).

### 2.8. Safety Assessment In Vivo: Histology and Immune Activation

**Figure 9:**
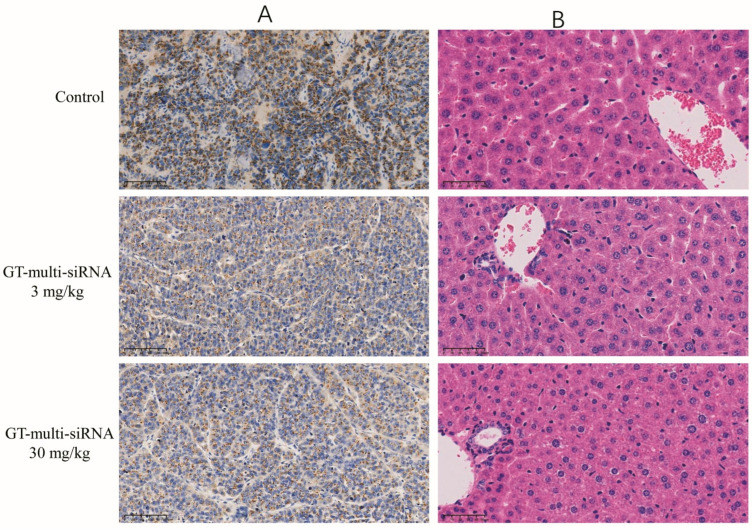
Immunohistochemistry analysis of tumors (**A**) and H&E staining of liver (**B**) in the treated mice. The scale bars = 100 μm.

**Figure 10:**
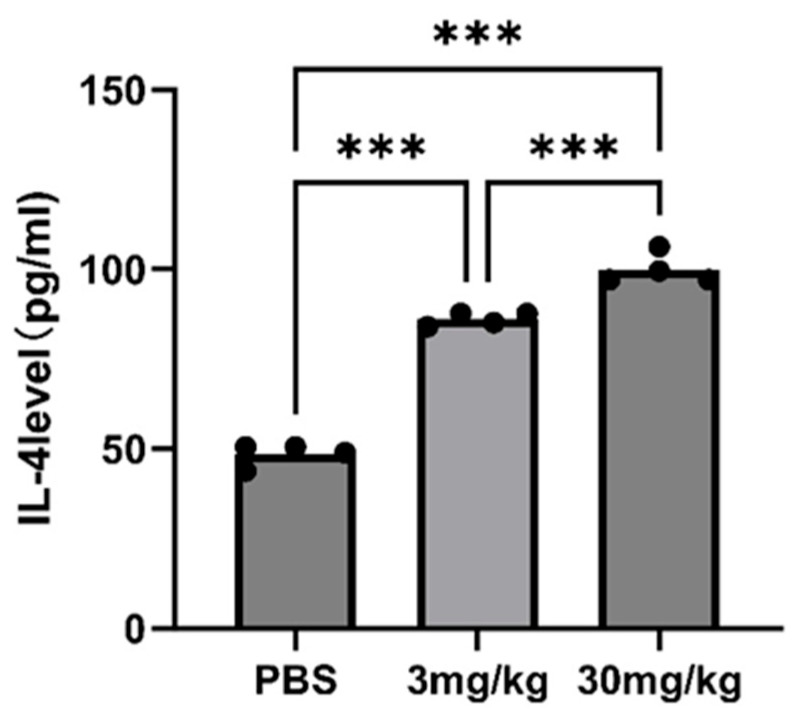
Quantitative detection of IL-4 in multi-siRNA-inoculated mice with different doses (3 mg/kg and 30 mg/kg). Asterisks indicate a significant difference (*** *p* < 0.001).

An immunohistochemical examination was conducted to elucidate the antitumor mechanism of GT-multi-siRNA by analyzing the expression of the *GP73* proteins in tumors. As shown in Figure 9A, the expression of *GP73* in GT-multi-siRNA-treated groups (3 mg/kg and 30 mg/kg) was significantly down-regulated than that in the PBS-treated group (control). Moreover, higher concentrations of GT-multi-siRNA (30 mg/kg) inhibited the expression of *GP73* protein more significantly than lower concentrations of GT-multi-siRNA (3 mg/kg). These results indicated that GT-multi-siRNA effectively delayed tumor growth by targeting *GP73* and *hTERT*. H&E staining was used to analyze the toxicity of GT-multi-siRNA in the liver of mice. From Figure 9B, no obvious histological changes were found among different groups, which further suggests that GT-multi-siRNA is a promising prodrug for the treatment of HCC with little toxicity. In addition, the levels of serum interleukin-6 (IL-6) and IL-4 were measured in multi-siRNA-inoculated mice to evaluate the immune activation effect of multi-siRNA. The results showed that the level of IL-6 had no effect, but the level of IL-4 was up-regulated. Compared with the PBS control group, IL-4 was up-regulated by 1.5 times in the low dose group and 1.8 times in the high dose group (Figure 10).

## 3. Discussion

Efficient delivery systems are needed to improve RNAi therapy through mitigating the shortcomings of siRNA involving their easy degradation in serum, difficulties in crossing the cell membranes, or short half-life [31,32]. For example, recent work on mesoporous silica nanoparticle systems and breast-cancer-targeting nanoparticle-based siRNA carriers provides new design strategies [33]. Positively charged polymers, peptides, and lipids have been widely utilized to formulate siRNAs into a compact nanoparticle, facilitating the intracellular uptake of siRNAs [34]. For example, Phadke et al. used liposomal complex to deliver STMN1-targeted shRNA into colorectal tumor, which can reduce tumor growth by 44% at 7 days post-treatment [35]. Jiang et al. used PEIHA as a delivery vector for VEGF-targeted siRNA and achieved tumor inhibition through intratumoral injection once every 3 days [36]. Pan et al. developed a peptide vector, STR-HK, to deliver BCL-2-targeted siRNA and achieved high anti-tumor activity through the downregulation of the Bcl-2 protein in mice [37]. Cao et al. developed a PEG-PEI nanoparticle to deliver EGFR-targeted siRNA for lung cancer and the tumor almost disappeared on the 21st day after a single intratumoral injection [38]. Wang et al. injected cholesterol-conjugated siUCID-1 intratumorally three times a week for two weeks [39]. After 20 days, tumor growth was significantly inhibited in xenograft model mice. Lee et al. used ECO (1-aminoethylimino[bis(N-oleoylcysteinylaminoethyl) propionamide]) NPs to deliver DNApk-cs-targeted siRNA with daily intratumoral injections for 5 consecutive days, combined with radiotherapy, and achieved significant results in the treatment of gliomas [40]. Liu et al. developed a halloysite nanotube (HNT) to deliver RIPK4-targeted siRNA that can inhibit bladder cancer tumorigenesis through intratumoral injection once a week [41]. Piao et al. developed a PLGA-encapsulated siRNA nanoparticle and then was intratumorally injected into the MCF-7 xenograft mouse model and the tumor growth was significantly inhibited via a single treatment [42]. Chen et al. delivered the BCL-2-targeted siRNA with an in vivo transfection reagent to achieve significant inhibition of glioma cell growth [43]. In this study, a structural RNAi system was developed by using a polymerized and branched siRNA, named as GT-multi-siRNA which is a high molecular weight siRNA through connecting multiple *GP73*- and *hTERT*-targeted siRNAs to improve their stability and structural density, thereby achieving the effect of enhancing the uptake of cells and prolonging the duration of gene silence in RNAi-based gene therapy of liver cancer. Anti-HCC analysis showed that GT-multi-siRNA without an extra delivery carrier could also enter Hep3B liver cancer cells to silence the *GP73* and *TERT* genes and inhibit Hep3B cell proliferation and migration. Moreover, GT-multi-siRNA can effectively inhibit the growth of tumors with low toxicity in a tumor model. These results confirmed that GT-multi-siRNA can effectively inhibit tumor growth within 15 days after a single injection. This single-dose efficacy profile aligns with emerging liver-targeted gene therapies, such as adeno-associated virus (AAV)-mediated delivery systems. For instance, Rajapaksha et al. demonstrated that a single intraperitoneal injection of AAV-ACE2 therapy achieved sustained (>3 months) inhibition of biliary fibrosis in murine models by shifting the hepatic angiotensin peptide balance [44]. Compared to viral vector-based approaches like AAV, GT-multi-siRNA offers distinct advantages: (1) It eliminates the need for viral carriers, mitigating immunogenicity risks; (2) Its branched RNA structure enables simultaneous multi-gene silencing without complex vector engineering; (3) Scalable production in *E. coli* provides a cost-effective alternative to viral manufacturing. While AAV excels in long-term expression (e.g., >80% fibrosis reduction at 9 months post-injection), GT-multi-siRNA provides a rapid, non-viral platform suitable for acute oncogene silencing in HCC However, the inhibitory ability of GT-multi-siRNA on tumor growth was decreased after 9 dpt. The potential reason is that before 9dpt, siRNAs will be gradually released from GT-multi-siRNAs under the processing of Dicer, thereby continuously inhibiting the level of target mRNA. After 9 dpt, the GT-multi-siRNAs may have been consumed, resulting in reduced inhibition efficiency of target genes and a rebound in tumor growth rate. To confirm the above speculation, we conducted another anti-tumor experiment of GT-multi-siRNA. Different from the previous single injection, in the second anti-tumor experiment, GT-multi-siRNA will be injected twice into tumor mice. The second GT-RNA injection was performed on the eighth day after the first injection. The results showed that on the 21st day, the tumor volume in the control mice had reached 2000 mm^3^, while the tumor volume in the mice injected with GT-multi-siRNA twice did not exceed 300 mm^3^ (Figure 8D), which was even smaller than the tumor volume of mice on the 15th day after a single injection.

Direct intratumoral injection of solid tumors with chemotherapy has the potential to overcome many limitations of conventional intravenous administration including severe toxicities resulting from systemic distribution [45,46]. In RNAi therapy of tumor treatment, intratumor injection is also one of the most common methods. For example, Ye et al. studied the antitumor effect of hCHST15-targeted siRNA in KPC-implantation model mice by intratumoral injection of naked siRNA twice a week [47]. Intratumoral injection once or more a week has also been used to analyze the antitumor effects of adeno-associated viruses expressing various antitumor siRNAs [48,49,50,51,52,53,54,55,56,57]. Shibata et al. directly intratumoral injected a plasmid expressing VEGF-C-siRNA into tumors every three days to achieve anti-tumor therapy in mouse models [58]. André et al. effectively inhibited the growth of melanoma tumors in model mice by a single intratumoral injection of CXCR4-shRNA expression plasmids [59]. In this study, we also used intratumoral injections to deliver the GT-multi-siRNA targeting both GP73 and hTERT to HCC tumors. After 15 days of a single intratumoral injection, tumor growth was significantly inhibited in the xenograft mouse. Moreover, the anti-tumor effect of GT-multi-siRNA will be more significant if another intratumoral injection is performed on the 8th day after the first injection. The results of immunohistochemistry showed that GT-multi-siRNA had little toxicity or harm to the mouse.

The effectiveness of RNA interference in cancer therapy has the following characteristics including high efficiency and potential, low cost compared with other gene therapy methods [60], and higher specificity compared with traditional cancer therapy such as chemotherapy. Therefore, cancer therapy is one of the main applications of RNAi-based therapy. Oncogenes, mutated tumor suppressor genes, and other genes involved in tumor progression are good targets for RNAi-based gene silencing because of the precise functional mechanism, high potential, and high specificity of RNAi, and lack side effects compared to chemotherapy [61]. In this study, both *GP73* and *hTERT* were selected to design GT-multi-siRNA, a dual gene-targeted multi-siRNA with the branched structure of RNAi therapy for liver cancer. Anti-tumor analysis showed that GT-multi-siRNA can effectively inhibit tumor growth within half a month after only one injection. High expression of *GP73* is highly related to HCC, but no targeted drugs for *GP73* have been released so far [23]. There are research reports that *GP73* regulated oncolytic adenovirus exhibits significant anti-tumor efficacy in hepatocellular carcinoma [62]. Therefore, this study lays the foundation for the development of targeted drugs targeting *GP73*.

## 4. Materials and Methods

### 4.1. Cell Lines

The HCC cell line (Hep3B) was kindly provided by Prof. Yigang Wang, Zhejiang Sci-tech University (Hangzhou, China). The vero cell line was kindly provided by Prof. Yulong He, Zhejiang Sci-tech University (Hangzhou, China). *E. coli* strain HT115 was maintained by our laboratory.

### 4.2. Cell Culture and Medium

Hep3B and vero cells were cultured in the Dulbecco’s Modified Eagle Medium (DMEM) (Biosharp, Hefei, China) containing 10% fetal bovine serum (FBS, Noverse, Germany), 100 U/mL penicillin, and 0.1 mg/mL streptomycin (Beyotime, Shanghai, China). LB medium was used for the cultivation of *E. coli*.

### 4.3. Design of a Dual Gene-Targeted Multi-siRNA with Branched Structure

SiRNAs targeting GP73 (NM_177937.3) and hTERT (NM_001193376.3) were designed using the DSIR platform, selecting candidates with scores above 90 and no more than three identical consecutive nucleotides [63]. Potential off-target interactions were evaluated through BLASTn (version. 2.12.0) searches against the human genome, using default settings with the word size set to 7. After removing sequences with off-target matches, the selected siRNAs were linked using defined hairpin loops (5-UCGG-3, 5-AACAGGUGA-3, and 5-AACACCUGA-3) capable of converting the ends of double-stranded siRNAs into single-stranded stem-loop structures. Tetra-U helix motifs were incorporated to connect three duplex segments and generate a branched, Y-shaped RNA configuration [64]. The secondary structure of the resulting high–molecular-weight molecule was predicted using RNAfold, and the construct was designated GT-multi-siRNA.

### 4.4. Biosynthesis of the GT-Multi-siRNA

To enable transcription, the DNA fragment encoding GT-multi-siRNA was first engineered to contain a T7 promoter at its 5 end (5-TCTAATACGACTCACTATA-3). Restriction sites for Bgl II and Bpu 1102 were then added to both termini of the sequence. The finalized construct was chemically synthesized by Edxinli Technology Co., Ltd. (Shaoxing, China) and cloned into the pET28a vector for expression of GT-multi-siRNA in *E. coli*. The recombinant plasmid was transformed into *E. coli* HT115 (DE3), and RNA expression was initiated with 1 mmol/L IPTG when the culture reached an OD600 of 0.4. After 4 h of induction, 1 mL of the bacterial culture was collected and resuspended in 100 µL of RNase-free Tris–Mg buffer (10 mmol/L Tris-HCl, pH 8.0; 10 mmol/L MgSO_4_). Subsequently, a mixture of phenol: chloroform: isoamyl alcohol (25:24:1) was added in equal volume to lyse the cells. The aqueous component of the total cell lysates was purified with 1/2 volume of chloroform to eliminate any residual phenol. The resulting aqueous solution containing GT-multi-siRNA was then directly subjected to examination via agarose gel electrophoresis.

### 4.5. Dynamic Light Scattering and Zeta Potential

GT-multi-siRNA (1 μg/μL in RNase-free water) was analyzed using a Zetasizer Nano ZS (Malvern Instruments, Worcestershire, UK). Average hydrodynamic diameter and zeta potential were measured in triplicate at 25 °C.

### 4.6. Stem-Loop RT-PCR

Total cellular miRNA was extracted using MiPure Cell/Tissue miRNA Kit (Vazyme, Nanjing, China) from Hep3B cells and treated with RNase-free DNase. MiRNAs were reverse-transcribed by reverse transcriptase at 37 °C using 0.2 µM stem-loop primers specific to hTERT-siRNAs (Table S1). For PCR amplification, 2 µL of cDNA template was combined with 12.5 µL of 2 × PCR mix (Takara, Shiga, Japan), 1 µM specific primers, and ddH_2_O to reach a final volume of 25 µL. PCR conditions included an initial denaturation at 95 °C for 10 s, followed by 35 cycles of denaturation at 95 °C for 10 s, annealing at 60 °C for 30 s, and extension at 72 °C for 5 s. Amplified products were analyzed using non-denaturing polyacrylamide gel electrophoresis. Cell toxicity analysis of GT-multi-siRNA using MTT method.

Vero cells were plated in a 96-well plate at 5000 cells per well (Corning, Corning, NY, USA) in 1 mL culture medium and cultured for 24 h. Then, GT-multi-siRNA was added into each well with increasing concentrations of 100 ng/μL, 200 ng/μL, 500 ng/μL and 1000 ng/μL. Each concentration was repeated 6 times. The cells were incubated with GT-multi-siRNA solution for 48 h at 37  °C. The cells were incubated in 20 μL PBS solution without GT-multi-siRNA was used as the control. Cell viability was measured using the MTT method [65]. Briefly, MTT was added to each well with a final concentration of 0.5 mg/mL and then cultured at 37 °C for 4 h, a water-insoluble formazan produced by viable cells was solubilized with 150 µL DMSO, and cell viability and growth over time were estimated by measuring the absorbance at 560 nm using FLU Ostar Omega (BMG Labtech, Ortenberg, Germany).

### 4.7. Anti-HCC Activity of GT-Multi-siRNA Using MTT Method

Hep3B cells were plated in 96-well plates at a density of 5 × 10^3^ cells per well in 1 mL culture medium and cultured for 24 h at 37 °C. Then, GT-multi-siRNA was added into each well with increasing concentrations of 50 ng/μL, 100 ng/μL, 150 ng/μL and 200 ng/μL. Each concentration was repeated 6 times. These Hep3B cells were incubated with GT-multi-siRNA solution for 72 h at 37 °C. Cell viability was measured using the MTT method as above.

### 4.8. Wound Closure Assays

Hep3B cells, either untreated or exposed to 200 ng/μL GT-multi-siRNA, were plated in 6-well plates and grown to approximately 90–100% confluence. A scratch was created in the monolayer using a pipette tip, followed by two PBS washes to remove detached cells. Images from identical regions of each well were captured at 0.24 and 48 h using a microscope (Nikon, Tokyo, Japan) [66].

### 4.9. Total RNA Extraction, RT-qPCR and Stem-Loop RT-qPCR

Total RNA was isolated using the Fast Pure Cell/Tissue Total RNA Isolation Kit V2 (Vazyme, Nanjing, China), followed by RNase-free DNase treatment and quantification with a NanoDrop ND-1000 spectrophotometer. For poly(A) RNA detection, 1 µg of total RNA was reverse-transcribed at 42 °C using SuperScript III Reverse Transcriptase (Invitrogen, Carlsbad, CA, USA) and 2.5 µM Oligo(dT18). For siRNA analysis, 1 µg of RNA was instead reverse-transcribed at 37 °C with stem-loop primers designed for GP73- and hTERT-specific siRNAs. A parallel reaction lacking reverse transcriptase was conducted as a control to confirm the absence of genomic DNA in subsequent steps. qPCR was performed using SYBR Green chemistry on a qTOWER3G real-time PCR system (Jena, Germany). For each reaction, 2 µL of cDNA was mixed with 12.5 µL of 2 × SYBR Green Master Mix (Takara, Shiga, Japan), 1 µM of the corresponding primers, and nuclease-free water to a final volume of 25 µL. The amplification program consisted of an initial step at 95 °C for 10 s, followed by 40 cycles of 95 °C for 10 s and 60 °C for 30 s. All samples were run in triplicate, with no-template and no-RT controls included. Ct values were automatically generated by the qTOWER3G system. Relative expression levels were calculated using the 2^−ΔΔCt^ method, where ΔΔCt = (Ct_target − Ct_reference)_treatment − (Ct_target − Ct_reference)_control [67]. Primer sequences are provided in Supplementary Table S1.

### 4.10. Antibodies and Western Blotting Analysis

Cells treated with GT-multi-siRNA were collected. After washing cells with ice-cold phosphate-buffered saline (PBS), lysis was performed on ice for 30 min using 1 mL of buffer containing 1% Nonidet *p*-40, 25 mM Tris-HCl, 150 mM NaCl, 10 mM EDTA (pH 8.0), and a 1:50 dilution of protease inhibitor cocktail. GP73 and hTERT proteins were resolved on 8% SDS–PAGE gels, with GAPDH run under the same conditions as a loading control. Following electrophoresis, proteins were transferred to nitrocellulose membranes and blocked for 3 h with 3% bovine serum albumin. Membranes were then incubated overnight at 4 °C with primary antibodies against GP73 (1:2000, HUABIO, Woburn, MA, USA) or hTERT (1:2000, HUABIO), washed, and further incubated with alkaline phosphatase–conjugated secondary antibodies (1:50,000, HUABIO) in TBST for 2 h. Protein bands were developed using NBT/BCIP substrate and subsequently scanned for analysis.

### 4.11. In Vivo Anti-Tumor Efficacy of GT-Multi-siRNA in Animal Xenograft Model

BALB/c Nude mice (female, 4 or 6 weeks old) were purchased from Slaccas (Shanghai, China) [68]. Hep3B cells were washed with PBS and centrifuged. 100 µL of cells (5 × 10^6^ cells) were injected into the right subcutaneous side of mice. Tumor formation in mice after a week. Tumor volume was measured every two days using a vernier caliper and calculated with the formula: Volume = (length × width^2^). Prior to intratumoral injection, tumors were visually identified and gently palpated under aseptic conditions to locate the injection site. In cases where multiple tumors were present in a single mouse, the largest tumor was consistently selected for treatment. The same tumor was used for injections at each time point to ensure consistency across the experimental groups. The antitumor activity analysis of GT-multi-siRNA was performed twice. For the first antitumor detection, eighteen tumor-bearing mice were randomly divided into three groups, with 6 mice in each group. Then, 100 µL of PBS solution and two different concentrations of GT-multi-siRNA (3 mg/kg and 30 mg/kg) were injected into different groups by a single intratumoral injection. Tumor volumes were monitored every two days throughout the experimental period. Mice were sacrificed on day 21 after the first treatment. The method of sacrificing mice is cervical dislocation and the dislocation of mice is carried out by pinching the toes reflex to confirm the disappearance of the mice’s vital signs. The tumors were weighed and photographed. Tumor and liver samples were sent to Haoke (Hangzhou, China) for HE staining and immunohistochemistry analysis. For the second antitumor analysis, ten tumor-bearing mice were randomly divided into two groups, with 6 mice in each group. Then 100 µL of PBS solution and GT-multi-siRNA (30 mg/kg) were intratumorally injected into different groups twice. The second injection will be performed on the 8th day after the first injection. Tumor volumes were monitored every two days throughout the experimental period. The mice would be killed by cervical dislocation when the tumor volume in the PBS group reached approximately 2000 mm^3^.

### 4.12. Hematoxylin and Eosin (H&E) Staining and Immunohistochemistry Analysis

Mouse tumors obtained from autopsy underwent two washes in PBS, followed by fixation with 2 mL of 4% paraformaldehyde [69]. Tumors and livers of mice were removed and sent to the Haoke company (Hangzhou, China) for HE staining and immunohistochemistry analysis. Briefly, the tumor or tissue samples were resected, fixed in 10% formalin overnight at 4  °C, and paraffin-embedded according to the standard protocol. Four-micrometer sections were processed for hematoxylin and eosin (H&E) staining. For immunohistochemical staining, tissue sections were first deparaffinized in xylene and rehydrated in graded ethanol. Antigen retrieval was performed in 1 mM EDTA (pH 8.0) for 10 min. Sections were blocked with 5% skim milk. Incubation was performed with a primary antibody (*hTERT*, 1:200, Huabio, Shenzhen, China) at 4 °C overnight, followed by the HRP-conjugated secondary antibody at room temperature for 1 h. Sections were counterstained with hematoxylin and mounted with neutral resin [70]. Images were taken with a microscope (Olympus, Tokyo, Japan). Negative results were denoted by a blue color, while weak positive, medium positive, and strong positive results were represented by light yellow, brown, and dark brown colors, respectively. Randomly selecting three positions from each group’s sections, the positive cell rate was calculated by Image J (version1.53t.)

C57BL/6 mice were randomly divided into three groups (*n* = 4 per group): phosphate-buffered saline (PBS) control, low-dose treatment (3 mg/kg), and high-dose treatment (30 mg/kg). All formulations were administered via intramuscular injection at a total volume of 100 μL. Seven days after the initial administration, blood samples were collected from the submandibular vein. Serum interleukin-6 (IL-6) and interleukin-4 (IL-4) levels were quantified using commercial cytokine detection kits (RuiXin, Quanzhou, China) according to the manufacturer’s instructions. Following the first blood collection, mice received a second dose using the same injection protocol. One week later, blood was collected again, and serum IL-6 and IL-4 concentrations were determined as described above.

### 4.13. Statistical Analysis

Statistical analysis was performed using GraphPad Prism (Version10.1.2) software (GraphPad, San Diego, CA, USA). IC50 determination was performed using non-linear regression analysis (log [concentration] vs. Control). The normality distribution was assessed using the Shapiro–Wilk test. A comparison between the two groups was made using Student’s *t*-test. One-way ANOVA followed by Dunnett’s test was used to determine whether there was a statistically significant difference in different independent groups compared with a control group.

## 5. Conclusions

This study demonstrates that GT-multi-siRNA, a dual-target polymerized branched RNA against GP73 and hTERT, can be efficiently biosynthesized in *E. coli* and self-assembles into uniform nanoparticles with high structural stability. The branched architecture confers enhanced resistance to enzymatic degradation and thermal stress, enabling prolonged intracellular retention without the need for additional delivery carriers. GT-multi-siRNA effectively enters tumor cells, induces potent RNAi-mediated silencing of GP73 and hTERT, and suppresses tumor cell proliferation and migration in vitro. In vivo, it achieves significant tumor growth inhibition in HCC models with minimal systemic toxicity and modest immune modulation. These findings indicate that polymerized branched RNAs are a robust, carrier-free RNAi platform capable of delivering sustained antitumor activity both in vitro and in vivo, representing a promising therapeutic strategy for liver cancer and potentially other malignancies.

## Data Availability

The original contributions presented in the study are included in the article/Supplementary Material. Further inquiries can be directed at the corresponding author.

## Supplementary Materials

The following supporting information can be downloaded at: https://www.mdpi.com/article/10.3390/ph18121844/s1, Table S1: Sequences used in this study. Table S2: Expression levels of hTERT and GP73 in 374 tumors and 50 normal tissues downloaded from TCGA dataset. Table S3: MTT assay for detecting the viability of vero cells at different concentration of GT-multi-siRNA.
