# Supplementary material for: Development of a Dual Gene-Targeted Multi-Sirna with Branched Structure and Its Role in the Therapy of Liver Cancer
Source: Pharmaceuticals (Basel). 2025 Dec 3;18(12):1844. doi: 10.3390/ph18121844 (PMC12736085; doi:10.3390/ph18121844)
Supplement: Supplementary file 1 [file pharmaceuticals-18-01844-s001.zip › Table S1.pdf]

siRNA name	Sense strand of siRNA	Antisense strand of siRNA
sihTERT-1	CGGAAGAGUGUCUGGAGCAAG	UGCUCCAGACACUCUUCCGGU
sihTERT-2	CCGGUGUACGCCGAGACCAAG	UGGUCUCGGCGUACACCGGGG
sihTERT-3	UCAGGAACACCAAGAAGUUCA	AACUUCUUGGUGUUCCUGAGG
sihTERT-4	GACGGUGUGCACCAACAUCUA	GAUGUUGGUGCACACCGUCUG
sihTERT-5	ACUGUUCAGCGUGCUCAACUA	UAGUUGAGCACGCUGAACAGU
siGP73	AGUGUGAGGAGCGAAUAGAAG	UCUAUUCGCUCCUCACACUGU

Primers of qPCR	Sequence (5'->3')
GP73_F	GTGTGAGGAGCGAATAGAAGAGG
GP73_R	GTCTCTGGTCGTTGTTTTCACT
hTERT_F	GCCGATTGTGAACATGGACTAC
hTERT_R	GCTCGTAGTTGAGCACGCTGAA
GAPDH_F	GAAAGCCTGCCGGTGACTAA
GAPDH_R	GCATCACCCGGAGGAGAAAT

Primers of stem-loop RT-PCR	Sequence (5'->3')
sihTERT-1-SL	GTCGTATCCAGTGCAGGGTCCGAGGTATTCGCACTGGATACGACCCGGAA
sihTERT-5-SL	GTCGTATCCAGTGCAGGGTCCGAGGTATTCGCACTGGATACGACGACGGT
sihTERT-1-F	CGCGTTGCTCCAGACACTC
sihTERT-5-F	GCGCGTAGATGTTGGTGCAC
sihTERT-R	AGTGCAGGGTCCGAGGTATT

polymerized siRNA with branched structure	Sequence (5'->3')
GT-multi-siRNA	GGGCAGAGUGGACCAUCAUUCCUUUUGCCAUAGCUGGAGUAGUCGCUCUGCACCGCAAGGUGCAGAGCGACUACUCCAGCUAUGGCUUUUGCUACCGGAAGAGUGUCUGGAGCAAGAGUGGACCAUCAUUCCUUUUGCCUAGAUGUUGGUGCACACCGUCUGGCUCCGGCCAGACGGUGUGCACCAACAUCUAGGCUUUUGUCCCCGGUGUACGCCGAGACCAAGAGUGGACCAUCAUUCCUUUUGCCUAGUUGAGCACGCUGAACAGUGCCCGCAAGGGCACUGUUCAGCGUGCUCAACUAGGCUUUUGCCUCAGGAACACCAAGAAGUUCAAAGGAGGCAUGAACUUCUUGGUGUUCCUGAGGCUUUUGGAAUGAUGGUCCACUCUUGGUCUCGGCGUACACCGGGGGACUUUUGGAAUGAUGGUCCACU

CUUGCUCCAGACACUCUUCCGGUAGCUUUUGGAAUG
AUGGUCCACUCUGCCCGGAUGGUCCACUCCUUACACU
UUUGGAAUGAUGGUCAAGCCUCCAGACCAUCAUUCC
UUUUGCCUUCUAUUCGCUCCUCACACUGUUCCUUCG
GGAACAGUGUGAGGAGCGAAUAGAAGGCUUUUGUGU
AAGGAGUGGACCAUCC

Table S1: Sequences used in this study.
