# Supplementary material for: Development of a Dual Gene-Targeted Multi-Sirna with Branched Structure and Its Role in the Therapy of Liver Cancer
Source: Pharmaceuticals (Basel). 2025 Dec 3;18(12):1844. doi: 10.3390/ph18121844 (PMC12736085; doi:10.3390/ph18121844)
Supplement: Supplementary file 1 [file pharmaceuticals-18-01844-s001.zip › Table S2 .pdf]

Table S2 Expression levels of hTERT and GP73 in 374 tumors and 50 normal tissues downloaded from TCGA dataset

Samples	Expression levels of hTERT		Expression levels of GP73	
	TPM	Log2 (TPM+1)	TPM	Log2 (TPM+1)
HCC-1	0.040178261	0.056830792	7.003475667	3.000626655
HCC-2	0.073567681	0.102413145	4.050120243	2.336317739
HCC-3	1.101485011	1.071409166	5.904630261	2.787564161
HCC-4	0.62720688	0.702397684	4.4198402	2.438250315
HCC-5	0.091215141	0.125935568	4.371556126	2.425340094
HCC-6	0.373372113	0.457722574	6.014499225	2.810340111
HCC-7	0.315328093	0.395422708	6.135466649	2.835007783
HCC-8	1.52876335	1.338432031	4.57251395	2.478328323
HCC-9	31.18314027	5.008233199	3.525912748	2.178208772
HCC-10	0	0	5.96266435	2.799639477
HCC-11	0.16816535	0.224244498	5.279194624	2.650579529
HCC-12	0.046097077	0.065016739	6.414838175	2.890415207
HCC-13	13.72903524	3.880591031	3.531917748	2.180121677
HCC-14	4.381932134	2.428124199	5.405173317	2.679237609
HCC-15	0.143474242	0.193423867	5.092209076	2.606965454
HCC-16	1.587593243	1.37161085	4.233109414	2.387668423
HCC-17	0.758588377	0.814417839	6.445923341	2.896450763
HCC-18	0.500744095	0.585677991	6.317745808	2.871399302
HCC-19	3.171387296	2.060527267	4.537994422	2.469363601
HCC-20	0.008003738	0.011500988	2.421337412	1.77456039
HCC-21	0.122087961	0.166185775	6.344330182	2.87663092
HCC-22	1.73262012	1.450284915	5.41259197	2.680907612
HCC-23	0.023220893	0.033117628	6.248299173	2.857642504
HCC-24	0.044161558	0.062344951	4.492499978	2.457462959
HCC-25	2.155789944	1.65800118	5.336735996	2.663739911

HCC-26	1.049793728	1.035478738	6.07939084	2.823625226
HCC-27	0.058196846	0.081608023	6.256688567	2.859311356
HCC-28	0.169411464	0.22578264	6.331999871	2.874206761
HCC-29	0.93878942	0.955156115	4.452177767	2.446832602
HCC-30	36.98107955	5.247209007	7.043411172	3.007807471
HCC-31	0.361080035	0.444751904	8.379755486	3.229550315
HCC-32	15.38199998	4.034039593	6.960570801	2.992871881
HCC-33	1.73817654	1.453215465	4.899080226	2.56049003
HCC-34	0.973331897	0.980633625	4.012319601	2.325478408
HCC-35	0	0	4.670758095	2.503541615
HCC-36	7.689200497	3.119223439	5.949043882	2.796814491
HCC-37	0.151394276	0.203381945	7.67960975	3.117630178
HCC-38	0.224999835	0.292781555	4.811883089	2.539005682
HCC-39	0.369015599	0.453138885	2.440057407	1.78243264
HCC-40	10.16798068	3.481296445	4.153887141	2.365660946
HCC-41	0	0	5.180057233	2.627620199
HCC-42	0.301142825	0.379779334	6.866803316	2.975777514
HCC-43	25.34482757	4.719447832	7.094092548	3.016869345
HCC-44	0.082386423	0.114215648	6.022749387	2.812035952
HCC-45	0.009688545	0.013910339	4.642329216	2.496290846
HCC-46	60.81846361	5.949965894	6.135258528	2.834965703
HCC-47	0.08617381	0.119254982	4.847821271	2.547899218
HCC-48	0.423658838	0.509603464	5.057315868	2.598678645
HCC-49	3.402397967	2.138289568	4.97495571	2.578928019
HCC-50	3.551185906	2.186242518	6.729668429	2.95040653
HCC-51	0.353258415	0.43643736	2.463285788	1.792141443
HCC-52	0.14129834	0.190675968	6.108940291	2.829634518
HCC-53	2.611228373	1.852489659	6.53127345	2.912893828
HCC-54	3.188490318	2.066430339	5.42295995	2.683238301

HCC-55	0.326387832	0.407502677	6.621408808	2.930057703
HCC-56	4.077767894	2.34419445	4.935503418	2.569370397
HCC-57	0.035990042	0.051010136	6.290154205	2.865949331
HCC-58	0.295862744	0.373912918	7.183437577	3.032706996
HCC-59	0.023052389	0.032880026	7.866950324	3.148437993
HCC-60	2.212290107	1.683602191	7.046187577	3.008305371
HCC-61	0.311154828	0.390838056	6.612397997	2.928350992
HCC-62	18.20752571	4.26359978	8.416741046	3.235227857
HCC-63	1.849539826	1.510728956	8.548282585	3.255241264
HCC-64	0.28325492	0.359807792	4.555172629	2.473831744
HCC-65	0.138876194	0.187610922	6.866633685	2.975746405
HCC-66	0.316431042	0.396631951	6.276855916	2.863315245
HCC-67	5.328356559	2.661830888	7.44945213	3.078857799
HCC-68	0.093670933	0.129178721	5.958341893	2.798743566
HCC-69	0.039062438	0.055282349	6.794308045	2.962420951
HCC-70	0.028657357	0.040762504	4.141118454	2.362082253
HCC-71	7.114618925	3.020523345	7.204897085	3.036485238
HCC-72	0.032462552	0.046089455	6.657748887	2.936920352
HCC-73	0.298679852	0.377045825	3.817412346	2.268258416
HCC-74	0.444422985	0.530493283	2.321633377	1.731892846
HCC-75	44.41688852	5.505156966	7.106627829	3.019101911
HCC-76	0.331965021	0.413556197	2.942697752	1.979183117
HCC-77	0.145307349	0.195734805	7.018674281	3.003363737
HCC-78	0	0	8.294857844	3.216432801
HCC-79	2.869884345	1.952290451	3.3404233	2.117835748
HCC-80	8.120706682	3.18914561	1.592820205	1.374522168
HCC-81	7.993637947	3.168904807	4.708545656	2.513123243
HCC-82	3.855510729	2.279623054	2.782670927	1.919405275
HCC-83	0.741249651	0.800123064	5.131496133	2.616239146

HCC-84	23.01844998	4.586071145	7.759388363	3.130830135
HCC-85	0.4451466	0.531215851	3.028478062	2.010234899
HCC-86	3.211874842	2.074462566	3.469926	2.160250948
HCC-87	0.245892003	0.317179017	5.028233178	2.591735223
HCC-88	1.204302473	1.140322203	2.847137176	1.943785272
HCC-89	0.013720273	0.019659608	5.74377506	2.753556416
HCC-90	30.68569091	4.985759569	7.737280209	3.127184259
HCC-91	0.059784301	0.083770662	6.132869014	2.834482481
HCC-92	64.19637081	6.026719753	3.664388634	2.221687998
HCC-93	0.131601967	0.178366589	4.42228253	2.438900287
HCC-94	0.013273515	0.019023657	4.929515726	2.567914282
HCC-95	0.097594477	0.134345127	2.099670164	1.632114706
HCC-96	0.151194255	0.203131298	4.512728617	2.462766581
HCC-97	0.023639443	0.033707643	3.06950018	2.024851612
HCC-98	0.659330645	0.730601393	2.329762628	1.735419334
HCC-99	3.819337244	2.26883476	6.31898272	2.871643139
HCC-100	0.354062713	0.437294558	3.421303837	2.144471881
HCC-101	0.278553952	0.35451304	7.711507417	3.122922381
HCC-102	64.90412297	6.042296818	7.244791467	3.043483005
HCC-103	0.142879622	0.192673454	4.640851078	2.495912849
HCC-104	3.742465535	2.245637289	2.96783123	1.988350663
HCC-105	1.645144136	1.403346338	6.05590956	2.818832069
HCC-106	0	0	5.742824963	2.753353147
HCC-107	0.261193221	0.33478932	6.541710402	2.914891753
HCC-108	0	0	5.940185629	2.794974251
HCC-109	3.179661996	2.063386278	5.487572335	2.697678719
HCC-110	0.010099306	0.014497135	6.392912727	2.886142882
HCC-111	0.053124833	0.074676457	4.761342144	2.526404937
HCC-112	0.423033454	0.508969578	2.898686559	1.962988172

HCC-113	0.282367753	0.358810053	5.658420387	2.73517996
HCC-114	0.641693095	0.715184449	6.794424354	2.962442479
HCC-115	0.216701003	0.282974678	6.604638371	2.926879644
HCC-116	0.169611762	0.226029724	5.507727459	2.702153833
HCC-117	0.113682895	0.155338505	1.477114647	1.308660642
HCC-118	3.334256406	2.115784503	6.357938184	2.879301556
HCC-119	0.193283155	0.254936422	6.613629323	2.928584333
HCC-120	0	0	3.392263319	2.134964547
HCC-121	14.71341348	3.973924711	3.814000118	2.267236177
HCC-122	1.78901713	1.479756795	5.201075108	2.632518364
HCC-123	0.38308788	0.467892826	3.807084509	2.265162167
HCC-124	0.798734783	0.846982482	5.920167683	2.790806996
HCC-125	0.009983462	0.01433167	9.681551723	3.417049339
HCC-126	0.498464779	0.583485174	6.857865618	2.974137496
HCC-127	0.159149093	0.213066141	6.226287113	2.853254577
HCC-128	0.045028318	0.063542036	5.536352751	2.708485844
HCC-129	0.130339417	0.176756049	5.384470655	2.674567009
HCC-130	2.693403844	1.884951018	6.292089337	2.866332236
HCC-131	0.011687991	0.016764425	8.242799044	3.208329817
HCC-132	0.126484265	0.171827162	3.831100198	2.272351775
HCC-133	0.121929917	0.165982558	5.59343888	2.721031115
HCC-134	0.271504211	0.34653624	1.833626014	1.502649362
HCC-135	0.838841258	0.878796941	6.561913614	2.918751368
HCC-136	0.515612547	0.599900988	3.574503789	2.193615258
HCC-137	0.257095045	0.330093732	5.985405254	2.804343815
HCC-138	1.353974741	1.23509884	5.78405825	2.762148557
HCC-139	1.535359145	1.342190126	8.15685575	3.194852296
HCC-140	0.04585866	0.064687896	7.033226158	3.005979493
HCC-141	4.345956514	2.418448102	5.495610024	2.69946502

HCC-142	0.263288068	0.337183655	4.055418478	2.337830521
HCC-143	1.076247306	1.053978296	6.346301895	2.877018185
HCC-144	1.572891717	1.363390741	6.099475363	2.827712416
HCC-145	0.021842187	0.031172404	2.601149816	1.84845762
HCC-146	0.055225249	0.07755099	2.248767114	1.699892329
HCC-147	16.88907225	4.161006664	2.528209102	1.818936066
HCC-148	2.345816875	1.742358485	4.259448012	2.394911394
HCC-149	0.032480407	0.046114405	4.269713615	2.39772456
HCC-150	15.67546576	4.059655153	6.705425051	2.945874542
HCC-151	0.139093066	0.187885623	4.853670319	2.549341495
HCC-152	0.293861027	0.371682666	5.397369135	2.677478731
HCC-153	0.094035865	0.129660033	5.120021339	2.613536683
HCC-154	0.671716859	0.741330517	5.419369375	2.682431577
HCC-155	0.560248554	0.641775875	4.566811217	2.476851159
HCC-156	0.309861315	0.389414071	2.561485437	1.832479091
HCC-157	0	0	5.760127164	2.757050385
HCC-158	0.417549769	0.503399388	5.66643524	2.736915512
HCC-159	0.954050345	0.966467638	1.948612071	1.560036029
HCC-160	0.022519646	0.032128562	2.69446735	1.885366379
HCC-161	7.103854499	3.018608271	4.251670631	2.392776438
HCC-162	0.124717712	0.169562951	8.561784603	3.257279907
HCC-163	0.010023582	0.014388977	6.046057369	2.816816221
HCC-164	0	0	5.244974319	2.642695639
HCC-165	0.012621708	0.018095317	7.088863504	3.015937016
HCC-166	0.012312298	0.01765443	3.479296254	2.163272087
HCC-167	0.16875495	0.224972475	5.699436621	2.744039779
HCC-168	6.438674381	2.895045547	2.558976265	1.831462311
HCC-169	0.323681263	0.404555769	3.930376437	2.301697801
HCC-170	0.084707763	0.117306411	4.989809392	2.582510094

HCC-171	0.194684237	0.256629355	5.395699996	2.677102268
HCC-172	0.111321995	0.152276885	5.63560902	2.730228883
HCC-173	0	0	5.946154317	2.796214462
HCC-174	0.363745142	0.447574057	3.610208622	2.204832037
HCC-175	0.11974937	0.163175855	2.655701536	1.870148289
HCC-176	0.10469931	0.143653733	6.797064419	2.962931054
HCC-177	0.367267443	0.451295467	6.768181032	2.957576822
HCC-178	1.183935999	1.126930578	7.006728423	3.001212873
HCC-179	2.482105881	1.799960072	3.808700921	2.2656472
HCC-180	0.081852165	0.113503369	7.057699708	3.01036804
HCC-181	0.02351606	0.033533739	6.557262931	2.917863818
HCC-182	0.032316719	0.045885663	4.619472498	2.490434711
HCC-183	0.588918425	0.668045059	8.328029468	3.221572346
HCC-184	0.690915405	0.757804485	6.350879859	2.877916943
HCC-185	0	0	4.247358541	2.39159137
HCC-186	0.367127457	0.451147752	4.77645127	2.530183453
HCC-187	5.904401381	2.787516337	4.251286737	2.392670974
HCC-188	6.463834295	2.899916957	5.533099225	2.707767552
HCC-189	0.808432703	0.854739912	2.426610407	1.776782172
HCC-190	0.062985469	0.088121876	3.497923363	2.16925908
HCC-191	6.033516494	2.814246163	3.887368459	2.289057874
HCC-192	0.205261758	0.269346504	5.755626996	2.756089674
HCC-193	0.426879916	0.512863925	5.040409595	2.594646381
HCC-194	0.016516268	0.023633303	5.368595095	2.670975151
HCC-195	0.10138047	0.139312931	5.942302605	2.795414252
HCC-196	2.015189999	1.592248915	4.827610105	2.542904357
HCC-197	0.061103327	0.085565149	5.693660867	2.742795459
HCC-198	0.073061537	0.101732813	4.870058622	2.553374911
HCC-199	0.173712836	0.231079478	6.14010759	2.835945814

HCC-200	0.057079951	0.080084497	6.399817901	2.887489769
HCC-201	0.463204148	0.54913107	5.411507742	2.680663663
HCC-202	0.012185415	0.017473591	4.450337037	2.446345446
HCC-203	0.514896515	0.599219245	3.582902373	2.196261553
HCC-204	0.046462391	0.065520464	4.241559711	2.389996173
HCC-205	0.146870494	0.197702489	5.760257363	2.757078171
HCC-206	2.259281776	1.704554083	5.404872501	2.679169852
HCC-207	2.637510619	1.86295146	5.734628816	2.751598433
HCC-208	1.293471158	1.197532765	4.217947632	2.383482465
HCC-209	0.153644888	0.206199205	3.0796635	2.02845016
HCC-210	0.580903494	0.660749301	3.175038672	2.061789561
HCC-211	0.50040808	0.585354938	3.849478507	2.277829614
HCC-212	0	0	5.753827571	2.755705347
HCC-213	0.034662444	0.049160169	6.077355001	2.823210287
HCC-214	0.019956672	0.028507868	4.12834289	2.358492726
HCC-215	56.13184277	5.836223159	2.960744252	1.985771548
HCC-216	0.009975547	0.014320363	1.735651402	1.451884403
HCC-217	0.069880955	0.097450278	6.31706247	2.871264576
HCC-218	22.41132269	4.549134541	6.372698988	2.882192856
HCC-219	0.1517308	0.203803547	6.176460431	2.843272454
HCC-220	0.316682068	0.396907028	2.845563812	1.943195133
HCC-221	0.053704523	0.075470367	3.904650563	2.294150354
HCC-222	0.386077134	0.471007544	6.356679371	2.879054715
HCC-223	0.294086764	0.371934348	4.511881159	2.462544783
HCC-224	1.167986672	1.116355887	2.891928525	1.960485215
HCC-225	26.33692486	4.772779058	4.963510592	2.576161865
HCC-226	0.030795028	0.043757483	4.911986624	2.563643005
HCC-227	0.176035468	0.233931571	4.418786167	2.437969717
HCC-228	0.657979616	0.72942627	7.578896512	3.100792088

HCC-229	3.751247005	2.248306211	5.280413841	2.650859627
HCC-230	2.336910005	1.738512778	5.63622182	2.73036211
HCC-231	5.343617023	2.665305675	2.19542441	1.67600756
HCC-232	0.226172758	0.294162258	4.075479374	2.34354409
HCC-233	0.11980112	0.163242527	4.647699597	2.497663353
HCC-234	0.311991062	0.391757892	5.264685101	2.647241993
HCC-235	0.277801128	0.353663318	2.249720067	1.700315449
HCC-236	0.326981972	0.408148771	2.458638896	1.790204395
HCC-237	0.133486022	0.1807666	6.599955632	2.925990996
HCC-238	0.053113402	0.074660798	5.642950746	2.731824218
HCC-239	0.088614681	0.122493398	6.826275468	2.968325891
HCC-240	3.160311558	2.056691573	5.598231933	2.722079491
HCC-241	0.707487837	0.771875302	5.444059841	2.687969891
HCC-242	0.431381667	0.517408407	6.596655425	2.925364384
HCC-243	0.215287639	0.281297817	7.769727136	3.132531955
HCC-244	0.4852352	0.570691413	4.316331214	2.410430989
HCC-245	0.243121996	0.313967885	1.831285673	1.501457322
HCC-246	0.135007142	0.182701376	5.101414938	2.609143847
HCC-247	0.492267941	0.577506599	5.630939947	2.729213389
HCC-248	1.871256706	1.521682321	6.897548795	2.981404946
HCC-249	0.046565635	0.065662793	3.330943616	2.11468139
HCC-250	0.240212979	0.310587892	5.758107597	2.75661932
HCC-251	0.091594805	0.126437434	9.05691118	3.330115367
HCC-252	1.916983968	1.544477457	3.408068371	2.140146601
HCC-253	0.020316909	0.02901732	4.755768835	2.525008651
HCC-254	0.254290857	0.326871933	7.454936871	3.079793983
HCC-255	0.375062085	0.459496759	4.632643419	2.493812143
HCC-256	44.01336909	5.492281644	6.458784685	2.898940581
HCC-257	0.010339349	0.014839942	5.063584661	2.600170936

HCC-258	0.167035886	0.222848925	5.391637086	2.676185495
HCC-259	0.039968417	0.056539715	3.174386274	2.061564106
HCC-260	0.501837887	0.586729093	5.86330542	2.778903557
HCC-261	0.508579654	0.593190874	5.4663752	2.69295722
HCC-262	3.01528404	2.005502047	3.837894452	2.274379295
HCC-263	1.385068554	1.254030734	6.100164755	2.827852502
HCC-264	3.594107156	2.199784507	6.89889164	2.981650231
HCC-265	0.242607966	0.313371208	6.170005569	2.841974239
HCC-266	0.057172892	0.080211337	4.646523351	2.497362852
HCC-267	0.466848149	0.552719528	5.372011218	2.671748807
HCC-268	83.36665219	6.398600949	5.133434307	2.616695112
HCC-269	0.459771214	0.545742278	3.772690895	2.254802903
HCC-270	0.528121816	0.611759554	5.898992954	2.786385787
HCC-271	0.097258465	0.1339034	6.021180637	2.811713645
HCC-272	0.01756037	0.025114389	6.125912404	2.833074749
HCC-273	0.16703662	0.222849832	6.792999806	2.96217878
HCC-274	61.3502361	5.962323118	2.953693304	1.983200963
HCC-275	0.247089237	0.318564703	5.879833155	2.782373578
HCC-276	0.136597378	0.184721293	7.870408562	3.149000555
HCC-277	0.013236065	0.018970334	6.069943346	2.821698654
HCC-278	0.376794022	0.461312738	5.752202394	2.755358149
HCC-279	0.071897445	0.100166881	5.810175303	2.767691936
HCC-280	0.108003002	0.14796179	2.676128382	1.878187151
HCC-281	1.299891528	1.20156582	4.531766528	2.467740268
HCC-282	0.012247889	0.017562634	3.975647883	2.314884392
HCC-283	0.458765793	0.544748275	6.128336886	2.83356552
HCC-284	0.156276694	0.209486672	2.530182321	1.819742695
HCC-285	18.28124888	4.269126596	7.502094274	3.087818256
HCC-286	7.319842115	3.056556151	4.955323756	2.574179941

HCC-287	0	0	3.559706801	2.188941059
HCC-288	0.011389018	0.016338019	5.942545265	2.795464678
HCC-289	0.892844259	0.920555713	2.107848074	1.63591598
HCC-290	0.723171976	0.785066693	3.700337607	2.232764384
HCC-291	0.194196445	0.256040179	4.324855534	2.412742385
HCC-292	0.264580402	0.338658766	5.582369544	2.718607024
HCC-293	0.018037179	0.02579025	4.231608069	2.387254464
HCC-294	0.943739236	0.958834686	7.123854097	3.022164329
HCC-295	6.370504545	2.881763382	2.222062893	1.687984655
HCC-296	0.036060462	0.051108197	4.035799082	2.332220724
HCC-297	0.06711634	0.093717472	5.366579926	2.670518577
HCC-298	1.618379582	1.388674257	5.002582013	2.58558321
HCC-299	0.056286943	0.079001799	5.38319151	2.674277933
HCC-300	0.357533859	0.440988182	2.861962931	1.949334316
HCC-301	0	0	5.79470219	2.764410319
HCC-302	0.055988636	0.078594309	2.573135741	1.837190722
HCC-303	0.612860807	0.689621936	9.048517613	3.328910781
HCC-304	0.040938775	0.057885216	5.231856156	2.639661934
HCC-305	0.097603913	0.134357529	7.13571472	3.024269092
HCC-306	0.037817734	0.053553094	3.575974248	2.194078933
HCC-307	20.06390694	4.396701147	6.228824094	2.853760984
HCC-308	1.058083011	1.041301173	2.970667031	1.989381385
HCC-309	0.064587561	0.090294615	4.83616019	2.545019483
HCC-310	5.56184427	2.714101355	5.468933595	2.693527903
HCC-311	0.405326344	0.490905191	6.316294441	2.871113137
HCC-312	1.5144222	1.330226915	7.736806209	3.12710599
HCC-313	0.110337307	0.150998016	6.194945927	2.846983845
HCC-314	4.008147531	2.324277062	4.894600258	2.55939398
HCC-315	1.005556889	1.00400289	2.395906234	1.763796625

HCC-316	0.018820961	0.026900547	2.941050618	1.978580279
HCC-317	0.616559078	0.692926232	6.005284718	2.808443688
HCC-318	5.711136341	2.746557067	6.381933149	2.883998673
HCC-319	0	0	5.817779613	2.769301965
HCC-320	0.160965177	0.215324699	6.854404882	2.973501969
HCC-321	0.161088054	0.215477387	6.824004427	2.967907187
HCC-322	0	0	5.777678637	2.760791233
HCC-323	0	0	5.3228757	2.660580858
HCC-324	0.186818505	0.247099327	5.012896905	2.588060223
HCC-325	0.009895433	0.01420592	5.815721039	2.768866289
HCC-326	0.055015955	0.077264817	6.321967631	2.872231396
HCC-327	0.164872042	0.220171488	2.068071409	1.617332062
HCC-328	0.369395526	0.453539204	3.106667002	2.037967968
HCC-329	30.76271473	4.989262319	5.895229519	2.785598575
HCC-330	1.061349661	1.043589246	6.944149938	2.989892853
HCC-331	0.345767529	0.428429216	5.375241471	2.672479987
HCC-332	0.964401244	0.974089641	8.491692664	3.246665388
HCC-333	23.79689619	4.632087645	3.419262009	2.143805468
HCC-334	1.041367035	1.0295356	6.545351978	2.915588202
HCC-335	0.068685372	0.095837176	7.103525774	3.018549749
HCC-336	0.151188844	0.203124516	7.555111635	3.09678668
HCC-337	3.783460874	2.258054798	1.957122255	1.564193889
HCC-338	0.244707196	0.315806404	6.087888673	2.825355944
HCC-339	0.443268615	0.529339833	4.305227099	2.407414509
HCC-340	0.13042019	0.176859138	5.17108717	2.625524674
HCC-341	0.040806013	0.057701201	2.490471913	1.803422103
HCC-342	1.020574548	1.01476558	5.58124534	2.718360605
HCC-343	3.964251462	2.311576196	7.228314726	3.040596977
HCC-344	1.094461382	1.066579284	5.678472966	2.739518268

HCC-345	0.11176091	0.152846562	5.899857505	2.786566568
HCC-346	0.048306162	0.068060123	5.422848789	2.683213332
HCC-347	9.366685509	3.373882797	3.440743841	2.150801353
HCC-348	0.008677351	0.012464768	5.456580902	2.690770384
HCC-349	30.16739852	4.961965836	3.690905368	2.229866397
HCC-350	1.4751834	1.307535426	4.204929382	2.37987859
HCC-351	2.298411192	1.721771261	4.860938293	2.551131648
HCC-352	0.599799274	0.677890902	5.396334513	2.67724539
HCC-353	0.517826539	0.602006926	5.591920661	2.720698879
HCC-354	2.105295334	1.634730485	6.708629795	2.946474445
HCC-355	0.023159981	0.033031742	3.100657136	2.035855122
HCC-356	4.749395027	2.523410158	5.947319809	2.796456511
HCC-357	0.026805268	0.038162602	4.789634808	2.53347235
HCC-358	0.672527415	0.742029859	5.637728509	2.730689623
HCC-359	7.055615901	3.009994896	3.172408597	2.060880445
HCC-360	0.682318286	0.750450682	2.282028957	1.714587968
HCC-361	0.812357427	0.857867507	5.215023031	2.635759737
HCC-362	4.018471688	2.327248076	7.661687211	3.114648075
HCC-363	2.566381291	1.834460953	7.568477884	3.099038945
HCC-364	2.440186284	1.782486688	5.639720241	2.731122456
HCC-365	0.021460259	0.030633075	5.419720462	2.682510478
HCC-366	0.190005067	0.250967716	4.201913567	2.379042428
HCC-367	7.866106197	3.148300643	4.945795576	2.571869862
HCC-368	39.34564828	5.334341166	7.224574481	3.039941039
HCC-369	0.322620199	0.40339884	2.167707345	1.663439055
HCC-370	20.55085125	4.429672951	6.453589505	2.897935367
HCC-371	4.899138597	2.560504305	6.05600293	2.81885116
HCC-372	0.220968824	0.288026364	2.974038935	1.990606007
HCC-373	0.166873356	0.22264799	3.155529485	2.055032312

HCC-374	0.035897075	0.050880666	5.877141071	2.78180894
normal-1	0	0	2.560399082	1.832038961
normal-2	0	0	3.781886461	2.257579876
normal-3	0	0	3.452299842	2.154550755
normal-4	0	0	3.788237636	2.259494754
normal-5	0	0	5.016516242	2.588928362
normal-6	0	0	4.431874826	2.441450234
normal-7	0	0	2.878135375	1.955363165
normal-8	0	0	4.276035399	2.399454244
normal-9	0	0	4.116462672	2.35514673
normal-10	0	0	4.445856439	2.445158951
normal-11	0	0	3.66605255	2.222202555
normal-12	0.009416081	0.013520975	3.930041171	2.301599695
normal-13	0.010346371	0.014849968	2.963088356	1.986625132
normal-14	0	0	6.019889075	2.811448234
normal-15	0	0	3.723742313	2.239930266
normal-16	0	0	2.548035016	1.827020248
normal-17	0.021074482	0.030088106	4.630736194	2.493323561
normal-18	0	0	3.519739026	2.176239473
normal-19	0.020553288	0.029351515	4.516257597	2.463689829
normal-20	0.013658383	0.019571526	4.111353233	2.353705296
normal-21	0.017144216	0.024524246	3.763410921	2.25199501
normal-22	0	0	4.380727141	2.427801149
normal-23	0.028602171	0.040685104	3.624926326	2.209430384
normal-24	0	0	5.028301585	2.591751594
normal-25	0.059659406	0.083600631	3.020722463	2.007454755
normal-26	0	0	2.281703962	1.714445101
normal-27	0	0	4.814693471	2.53970314
normal-28	0	0	3.245704134	2.086003839

normal-29	0.011166808	0.016021012	4.991434008	2.582901343
normal-30	0	0	3.313351833	2.108809399
normal-31	0	0	2.067761209	1.617186189
normal-32	0	0	3.588558068	2.198040865
normal-33	0	0	3.616652924	2.206847276
normal-34	0	0	2.279340795	1.713405837
normal-35	0	0	3.427940814	2.146635939
normal-36	0	0	3.580136798	2.195390689
normal-37	0.012083862	0.017328838	2.222036647	1.687972903
normal-38	0	0	1.830124562	1.500865552
normal-39	0	0	3.399915718	2.137475889
normal-40	0	0	4.268955017	2.397516863
normal-41	0	0	3.024198274	2.008701389
normal-42	0	0	2.469235428	1.794617748
normal-43	0	0	5.804329635	2.766453034
normal-44	0.007691244	0.011053666	4.355395398	2.420993096
normal-45	0	0	5.191128247	2.630202345
normal-46	0.017241874	0.024662755	3.301442791	2.10482065
normal-47	0	0	2.978304239	1.99215361
normal-48	0	0	3.471124733	2.160637794
normal-49	0	0	4.480969938	2.454431222
normal-50	0	0	2.677880674	1.878874673
