# Supplementary material for: Development of a Dual Gene-Targeted Multi-Sirna with Branched Structure and Its Role in the Therapy of Liver Cancer
Source: Pharmaceuticals (Basel). 2025 Dec 3;18(12):1844. doi: 10.3390/ph18121844 (PMC12736085; doi:10.3390/ph18121844)
Supplement: Supplementary file 1 [file pharmaceuticals-18-01844-s001.zip › Table S3.pdf]

Table S3 MTT assay for detecting the viability of vero cells at different concentration of GT-multi-siRNA

Repeats	0 ng/μl	100 ng/μl	200 ng/μl	500 ng/μl	1000 ng/μl
repeat-1	1.005874	1.018055	0.966287	0.609587	0.047615
repeat-2	0.954245	1.054182	0.963380	0.633672	0.040556
repeat-3	1.026914	1.028990	0.928638	0.672844	0.040971
repeat-4	1.047399	1.089340	0.942895	0.610833	0.036265
repeat-5	0.976668	1.055289	0.966287	0.631319	0.039449
repeat-6	1.037572	1.027883	0.917841	0.666061	0.039864
repeat-7	1.057227	1.090724	0.977361	0.612909	0.036127
repeat-8	0.975284	1.058334	0.953553	0.634918	0.038757
repeat-9	1.031897	1.027883	0.943864	0.663155	0.041248
